# Supplementary material for: Clinical Evaluation of the VirClia IgM/IgG Chemiluminescence Tests for the Diagnosis of Tick-Borne Encephalitis in an Endemic Part of Norway
Source: Viruses. 2024 Sep 23;16(9):1505. doi: 10.3390/v16091505 (PMC11437423; doi:10.3390/v16091505)
Supplement: Supplementary file 1 [file viruses-16-01505-s001.zip › viruses-3219041-supplementary.pdf]

Table S1. Distribution of the test result combinations in the first serum sample among 85 TBE cases.

**Test Result Combination**	**n**	**%**
VirClia IgM, ReaScan IgM and VirClia IgG all positive	70	82.3
VirClia IgM negative, ReaScan IgM and VirClia IgG positive	3	3.5
VirClia IgM and ReaScan IgM negative, VirClia IgG positive	3	3.5
VirClia IgM and ReaScan IgM positive, VirClia IgG negative	5	5.9
VirClia IgM negative, ReaScan IgM positive and VirClia IgG negative	2	2.4
VirClia IgM, ReaScan IgM and VirClia IgG all negative	2	2.4
